# The Co-Evolutionary Arms Race Between *Salmonella* and the NLRC4 Inflammasome: Immune Recognition and Evasion Strategies

**DOI:** 10.3390/microorganisms14071500

**Published:** 2026-07-09

**Authors:** Yaxin Guo, Ruohan Chen, Yan Qian, Ying Xu, Chao Yin, Xinan Jiao, Zhiming Pan

**Affiliations:** 1 School of Public Health, Faculty of Medicine, Yangzhou University, Yangzhou 225000, China; h15662517147@163.com (R.C.); qy86330763@163.com (Y.Q.); 2 Jiangsu Co-Innovation Center for Prevention and Control of Important Animal Infectious Diseases and Zoonoses, Yangzhou University, Yangzhou 225000, China; jiao@yzu.edu.cn; 3 Jiangsu Key Laboratory of Zoonosis, Yangzhou University, Yangzhou 225000, China; 17384487022@163.com (Y.X.); chaoyin@jit.edu.cn (C.Y.); 4 Key Laboratory of Prevention and Control of Biological Hazard Factors (Animal Origin) for Agrifood Safety and Quality, Ministry of Agriculture of China, Yangzhou University, Yangzhou 225000, China; 5 Joint International Research Laboratory of Agriculture and Agri-product Safety of the Ministry of Education, Yangzhou University, Yangzhou 225000, China

**Keywords:** *Salmonella*, inflammasome, NLRC4, caspases, NAIP, immune evasion

## Abstract

*Salmonella* is a globally significant foodborne intracellular pathogen, and invasive salmonellosis poses a major global public health threat. The NLR family CARD-containing protein 4 (NLRC4) inflammasome, a pivotal cytosolic innate immune sensor, specifically recognizes *Salmonella* flagellin and type III secretion system (T3SS) components via the NAIP (NLR family apoptosis inhibitory protein) family. Upon activation, it triggers pyroptosis, pro-inflammatory cytokine release, and infected intestinal epithelial cell extrusion, serving as a central pathway for host defense against *Salmonella* colonization and systemic spread. This work systematically summarizes the structural composition, activation mechanisms, post-translational modifications, and regulatory protein network of the NLRC4 inflammasome, and highlights the molecular mechanisms by which *Salmonella* evades NLRC4 surveillance through multiple strategies: transcriptional downregulation of immunogenic ligands, structural modification of T3SS components, secretion of effector proteins, and chemotaxis-virulence synergy. A comprehensive delineation of the co-evolutionary arms race between *Salmonella* and the NLRC4 inflammasome provides an integrated mechanistic framework for understanding host–pathogen immune interplay. Deciphering the mechanisms of bacterial immune evasion on this basis holds critical importance for identifying novel anti-infective targets and advancing translational preventive and therapeutic strategies against salmonellosis.

## 1. Introduction

*Salmonella enterica*, a major foodborne pathogen, is a Gram-negative, motile, hydrogen sulfide-producing, facultative intracellular bacterium that causes a wide range of diseases in humans and animals, from self-limiting gastroenteritis to severe systemic infections [1]. According to the World Health Organization (WHO), invasive non-typhoidal *Salmonella* (iNTS) infections represent a pressing global public health challenge, causing millions of clinical cases and tens of thousands of deaths annually worldwide [2]. The heaviest disease burden is concentrated in low- and middle-income countries, where inadequate sanitation and limited access to safe drinking water sustain the transmission of this enteric pathogen [3]. Following oral ingestion and transit through the gastrointestinal tract, *Salmonella* initially crosses the intestinal epithelial barrier via microfold cells to gain access to Peyer’s patches before invading macrophages and disseminating throughout the reticuloendothelial system to establish systemic infection [4]. The pathogenesis of *Salmonella* relies on an array of conserved virulence factors, among which the type III secretion system (T3SS) and flagellin serve central, non-redundant roles. During the early invasive phase, *Salmonella* pathogenicity island-1 (SPI-1)-encoded T3SS-1 is abundantly expressed to mediate bacterial entry into non-phagocytic intestinal epithelial cells [5,6]. Following internalization, the *Salmonella* pathogenicity island-2 (SPI-2)-encoded T3SS-2 is upregulated to support intracellular survival and replication within the *Salmonella*-containing vacuole (SCV) [7,8]. Meanwhile, flagellin, the principal structural subunit of bacterial flagella, mediates motility and chemotaxis and also functions as a key pathogen-associated molecular pattern (PAMP) recognized by host immune surveillance systems [9].

As the first line of host defense against intracellular pathogens, the innate immune system has evolved a sophisticated repertoire of pattern recognition receptors (PRRs) that detect conserved microbial structures referred to as PAMPs [10]. The NOD-like receptor (NLR) family, a major class of cytosolic PRRs, fulfills non-redundant functions in detecting intracellular bacteria such as *Salmonella* and orchestrating context-appropriate innate immune responses [11,12]. Upon ligand recognition, specific NLR family members oligomerize to assemble large multiprotein signaling platforms termed inflammasomes. These complexes drive the activation of cysteine proteases, which mediate the proteolytic maturation of pro-inflammatory cytokines and initiate pyroptosis, an inflammatory form of regulated cell death [13,14]. Inflammasome activation is governed by a well-established two-signal paradigm: a priming step (signal 1) drives transcriptional upregulation of core inflammasome components and pro-inflammatory cytokine precursors, while an activation step (signal 2) triggers assembly of functional inflammasome complexes and subsequent Caspase-1 activation [15,16,17]. This two-signal regulatory circuit acts as an essential immune checkpoint, preventing aberrant pathological inflammation while preserving the capacity to mount rapid, effective immune defenses against microbial invasion [18,19].

Among all canonical inflammasomes, the NLR family CARD-containing protein 4 (NLRC4) inflammasome—also known as IPAF (ICE-protease activating factor)—stands out for its unique ligand recognition mode and its non-redundant role in host defense against T3SS-expressing intracellular bacteria [20,21,22]. Unlike most inflammasomes, which detect PAMPs or host-derived danger-associated molecular patterns (DAMPs) via indirect signaling cascades, the NLRC4 inflammasome mediates direct, specific recognition of bacterial flagellin and T3SS structural components through an upstream family of innate sensors termed NLR family apoptosis inhibitory proteins (NAIPs) [23,24,25,26]. Upon activation, the NLRC4 inflammasome triggers pyroptosis, the release of mature Interleukin-1β (IL-1β) and Interleukin-18 (IL-18), and the extrusion of infected intestinal epithelial cells, collectively restricting *Salmonella* colonization and systemic dissemination [27]. Genetic ablation of NLRC4 or its downstream signaling components confers markedly increased susceptibility to *Salmonella* infection in mice, particularly at mucosal surfaces where initial invasion occurs [27,28,29]. Conversely, excessive NLRC4 activation during severe systemic infection can drive cytokine storm and life-threatening coagulopathy [30], highlighting the need for tight regulation to balance effective pathogen clearance with avoidance of immunopathology.

Over millions of years of co-evolution with the mammalian innate immune system, *Salmonella* has evolved elaborate, stage-specific immune evasion strategies to counteract NLRC4-mediated host defenses, giving rise to a dynamic “recognition–evasion” arms race that represents a central determinant of infection outcome [29,31,32,33]. While individual studies have dissected discrete aspects of NLRC4 activation or *Salmonella* immune evasion, a systematic, integrated synthesis of this co-evolutionary interplay remains lacking. In this review, we systematically delineate the structural architecture, activation mechanisms, post-translational modifications, and regulatory protein network of the NLRC4 inflammasome. We next summarize the multifaceted strategies employed by *Salmonella* to subvert NLRC4 surveillance, including transcriptional silencing of immunogenic ligands, structural modification of T3SS components, secretion of inhibitory effector proteins, and chemotaxis-virulence synergy. Finally, we highlight outstanding questions and outline future research directions in the field. Collectively, this work establishes a holistic mechanistic framework for understanding host–pathogen co-evolution and provides a theoretical foundation for developing novel anti-infective therapeutics and next-generation *Salmonella* vaccines.

## 2. Architecture and Activation Mechanisms of the NLRC4 Inflammasome

### 2.1. Structural Composition and Interspecies Diversity of NAIP/NLRC4

The NLRC4 protein possesses the canonical tripartite modular architecture that defines the NLR protein family. It is structurally organized into three core conserved domains: an N-terminal caspase activation and recruitment domain (CARD), a central nucleotide-binding and oligomerization (NACHT) domain, and a C-terminal leucine-rich repeat (LRR) domain [34]. The assembly and activation of the NLRC4 inflammasome are strictly dependent on NAIP, an upstream partner and fellow member of the NLR family. While NLRC4 itself lacks the capacity to directly bind PAMPs, the NAIP family serves as the primary cognate receptor for conserved bacterial structural proteins [23].

The NAIP family displays pronounced interspecies variation in gene copy number and ligand recognition profiles, which directly shapes the pathogen-sensing repertoire of the NLRC4 inflammasome [25]. Structurally, all NAIP family members share a conserved multidomain architecture: three tandem N-terminal baculoviral inhibitor of apoptosis protein repeat (BIR) domains, a central NACHT domain, and a C-terminal LRR domain. The BIR domains function as the key structural determinants governing ligand recognition, and individual NAIP paralogs have evolved tailored specificities for distinct bacterial PAMPs [35,36].

The mouse genome encodes 7 distinct *Naip* paralogs (*Naip1* to *Naip7*) that give rise to the NAIP1–NAIP7 proteins [14,37]. Among these, four paralogs have well-defined ligand specificities: NAIP1 specifically recognizes the T3SS needle protein that forms the extracellular tip of the T3SS injectisome [38]; NAIP2 detects the T3SS inner rod protein, a core basal body component spanning the bacterial periplasm [37]; NAIP5 and NAIP6 both target flagellin, the major structural subunit of bacterial flagellar filaments [23]. In contrast, the human genome harbors only one functional *Naip* gene that encodes hNAIP. Through alternative mRNA splicing, full-length human NAIP can recognize all three ligand classes—T3SS needle protein, T3SS inner rod protein, and flagellin—rendering it functionally equivalent to the full complement of mouse NAIP paralogs combined [26]. The mouse multi-paralog system enables fine-tuned ligand-specific regulation, while the single human NAIP delivers broader sensing coverage; notably, the ligand specificities of NAIP3, NAIP4, and NAIP7 remain uncharacterized to date. This remarkable interspecies difference in NAIP genomic organization represents a striking example of evolutionary divergence in innate immune pathogen surveillance.

### 2.2. Assembly and Downstream Effector Functions of the NLRC4 Inflammasome

In the resting state, NLRC4 adopts a compact auto-inhibited conformation, in which the LRR domain folds back onto the NACHT domain, thereby preventing spontaneous oligomerization [39]. This auto-inhibition is pivotal to avoid aberrant inflammasome activation [34]. Upon recognition of bacterial ligands by NAIPs, NLRC4 undergoes conformational changes that relieve this auto-inhibition, exposing the NACHT domain for oligomerization and the CARD for pro-Caspase-1 recruitment [36]. The adaptor protein ASC (apoptosis-associated speck-like protein containing a CARD), which harbors an N-terminal pyrin domain (PYD) and a C-terminal CARD, has long been the subject of debate regarding its requirement in NLRC4 inflammasome activation [40]. Early biochemical studies proposed an ASC-independent activation model: NLRC4 can directly recruit and interact with pro-Caspase-1 via homotypic CARD–CARD interactions and is sufficient to trigger limited Caspase-1 activation and substrate cleavage in the absence of ASC [41]. However, accumulating subsequent evidence demonstrates that ASC is indispensable for full physiological activation of the NLRC4 inflammasome: ASC mediates the formation of large oligomeric signaling specks, which markedly enhance the efficiency of Caspase-1 autoactivation, pro-inflammatory cytokine maturation, and robust pyroptosis [42]. This discrepancy stems largely from varying experimental systems: in vitro overexpression bypasses the need for ASC-dependent signal amplification, while ASC is essential for full, rapid NLRC4 inflammasome activation in endogenous cells and in vivo models. ASC dependence also varies by cell type—stronger in macrophages than in epithelial cells—explaining inconsistent reports in the field [43].

Subsequently, activated Caspase-1 mediates site-specific proteolytic cleavage of multiple downstream substrates, including the pro-inflammatory cytokine precursors pro-Interleukin-1β (pro-IL-1β) and pro-Interleukin-18 (pro-IL-18) and gasdermin D (GSDMD) [44]. Proteolytic processing removes the N-terminal pro-domains of pro-IL-1β and pro-IL-18 to generate their mature, biologically active isoforms [45]. Concurrently, Caspase-1 cleaves GSDMD at the conserved aspartic acid residue Asp275, liberating the N-terminal pore-forming domain (GSDMD-NT) from its C-terminal auto-inhibitory domain [46]. GSDMD-NT undergoes homo-oligomerization and inserts into the host plasma membrane, forming transmembrane pores with an inner diameter of approximately 10–16 nm that permit the passage of ions and small cytosolic molecules [47]. These pores serve as the primary conduit for the non-classical secretion of mature IL-1β and IL-18 [48,49], which play non-redundant roles in orchestrating local inflammatory responses, recruiting neutrophils to the site of infection, and priming adaptive immunity [50]. Meanwhile, sustained pore formation disrupts cellular osmotic homeostasis, ultimately triggering pyroptosis and the release of DAMPs that further amplify inflammatory cascades [51].

### 2.3. Post-Translational Regulation of NLRC4 Activation

**Figure 1:**
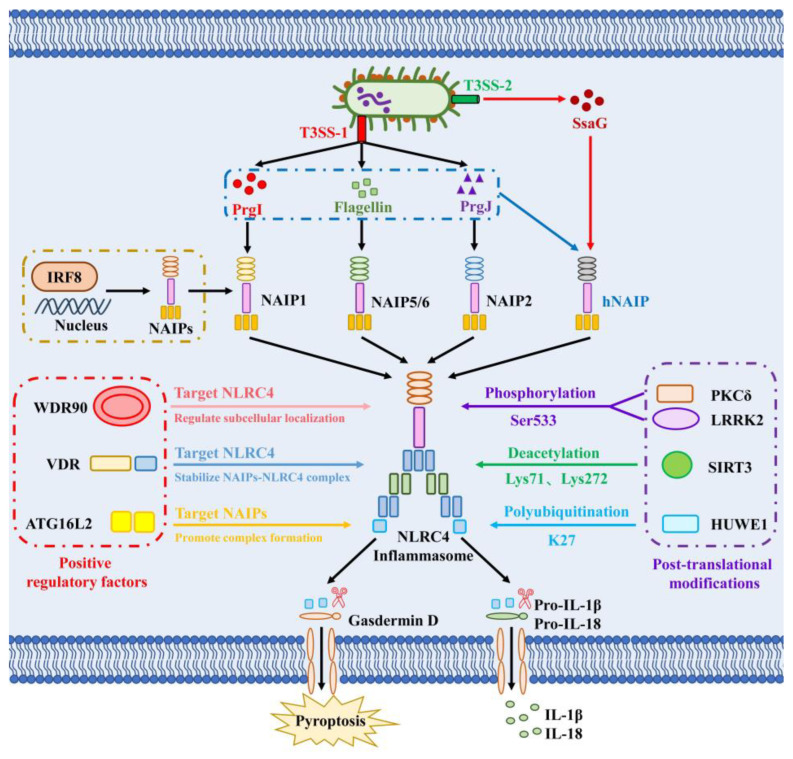
Recognition of *Salmonella* by the NAIP/NLRC4 inflammasome. Intracellular NLRC4 inflammasomes enable rapid detection of invading *Salmonella*, triggering pyroptosis and the release of pro-inflammatory cytokines IL-1β and IL-18. NAIPs act as the primary sensors for conserved bacterial PAMPs: in mice, NAIP1 specifically recognizes the T3SS-1 needle protein PrgI, NAIP2 targets the T3SS-1 inner rod protein PrgJ, and NAIP5/6 detect flagellin, whereas human NAIP (hNAIP) recognizes all three PAMPs. Importantly, hNAIP can also sense the T3SS-2 needle protein SsaG, which is essential for recognizing and eliminating *Salmonella* that have downregulated T3SS-1 and flagellin to disseminate systemically. Post-translational modifications of NLRC4 are indispensable for its full activation. PKCδ and LRRK2 catalyze phosphorylation of NLRC4 at Ser533. SIRT3 deacetylates NLRC4 at Lys71 and Lys272, promoting NLRC4 inflammasome activation by enhancing ASC speck formation and inflammasome complex assembly. The E3 ubiquitin ligase HUWE1 mediates K27-linked polyubiquitination of NLRC4 to facilitate its oligomerization, Caspase-1 recruitment, and inflammasome assembly. Beyond these modifications, several protein regulators positively modulate NLRC4 inflammasome activation and host defense against *Salmonella*. IRF8 directly binds to the promoter regions of *Naip* genes to control their transcription and support NLRC4 inflammasome activation. WDR90 interacts directly with NLRC4 to regulate its subcellular localization, thereby licensing inflammasome assembly and activation. The VDR functions as an upstream positive regulator by directly associating with NLRC4 to stabilize the NAIP–NLRC4 signaling complex. ATG16L2 binds directly to NAIPs, markedly enhancing ligand-induced NAIP–NLRC4 complex formation and subsequent inflammasome activation. Figure 1 was independently designed by the authors using Microsoft PowerPoint 2021, with all elements created using native software features.

The activation of the NLRC4 inflammasome is tightly regulated to ensure its proper immune function while avoiding host tissue damage caused by excessive inflammatory responses. Its core regulatory mechanisms mainly include post-translational modifications, transcriptional regulation, and the actions of dedicated regulatory proteins. Phosphorylation of NLRC4 at the Ser533 (S533) residue within its central NACHT domain represents an essential regulatory checkpoint for NLRC4 inflammasome activation [52]. This site-specific phosphorylation event is catalyzed by protein kinase Cδ (PKCδ) and leucine-rich repeat kinase 2 (LRRK2) (Figure 1) and is absolutely required for full activation of the NLRC4 inflammasome [41,53]. NLRC4 phosphorylation at Ser533 markedly enhances its capacity to recruit and activate Caspase-1, while phospho-deficient mutations at this site drastically impair inflammasome function [54]. Recent in vivo studies have further demonstrated that LRRK2-deficient mice exhibit severely blunted NLRC4 inflammasome activation and IL-1β secretion, alongside markedly increased susceptibility to *Salmonella* infection [55,56], highlighting the critical role of this regulatory phosphorylation event in antibacterial host defense. Notably, while both kinases converge on the same Ser533 phosphorylation site of NLRC4, they are triggered by distinct upstream signaling cues. This divergence in upstream activation enables the NLRC4 inflammasome to integrate a broad spectrum of danger signals across successive infection stages, endowing the regulatory network with an additional tier of functional complexity. Furthermore, acetylation of NLRC4 also acts as a key post-translational modification to regulate its activation. Sirtuin 3 (SIRT3), a mitochondrial-localized deacetylase, has been shown to deacetylate NLRC4 at Lys71 and Lys272 residues (Figure 1), thereby promoting NLRC4 inflammasome activation by enhancing ASC speck formation and inflammasome complex assembly [57]. Ubiquitination modifications exert bidirectional regulatory control over NLRC4 inflammasome activation. The E3 ubiquitin ligase HUWE1 mediates K27-linked non-degradative polyubiquitination of NLRC4, which promotes its oligomerization, Caspase-1 recruitment and subsequent inflammasome activation (Figure 1). Consistently, *Huwe1* deficiency impairs Caspase-1 activation and IL-1β production, leading to increased bacterial burden during *Salmonella* infection [58]. In contrast, two other E3 ligases, TRIM29 (tripartite motif containing 29) and HERC2, mediate K48-linked polyubiquitination and proteasomal degradation of NLRC4, acting as negative regulators to restrict excessive inflammasome activation [59,60]. These well-characterized regulatory nodes (phosphorylation and ubiquitination) provide actionable targets for translational development: selective NLRC4 agonists may serve as mucosal immune enhancers for enteric infection prevention, while specific inhibitors hold potential for mitigating cytokine storm and excessive immunopathology in severe invasive salmonellosis.

## 3. Mechanisms of NLRC4 Inflammasome-Mediated *Salmonella* Recognition and Clearance

### 3.1. Molecular Basis of Salmonella Ligand Recognition

Recognition of *Salmonella* by the NLRC4 inflammasome relies on the sensing of its specific PAMPs. The well-defined core ligands to date include flagellin and components of the T3SS [23]; these molecules are evolutionarily conserved during the pathogenesis of *Salmonella* and serve as key targets for host innate immune recognition. The T3SS and flagellar apparatus represent an Achilles’ heel for bacterial pathogens, which the host immune system has evolved to exploit. While essential for bacterial virulence, the structural components of the T3SS and flagella are exposed to the host cytosol during effector delivery, rendering them accessible to cytosolic immune sensors. Recognition of these components by the NLRC4 inflammasome is an elegant evolutionary strategy that turns the pathogen’s own virulence machinery against it. This mechanism is highly effective, as it targets conserved, functionally essential structural elements that the pathogen cannot easily modify to evade detection [32].

Flagellin is the major structural component of *Salmonella* flagella, which is required for bacterial motility and chemotaxis. Notably, NLRC4 is unable to directly sense intact extracellular *Salmonella* flagella; instead, it specifically recognizes monomeric flagellin that has gained access to the host cell cytosol, which is a prerequisite for subsequent NLRC4 inflammasome assembly and activation [61]. *Salmonella* mainly expresses two types of flagellin, FliC and FljB; despite sequence differences between the two proteins, both are sensed by murine NAIP5/6 and human NAIP to drive NLRC4 inflammasome activation [62,63]. *Salmonella* flagellin is mainly composed of four domains, namely D0, D1, D2 and D3. The D0 domain is highly conserved across *Salmonella* species and serves as the essential region for recognition by NAIPs [64]. Structural studies have revealed that the D0 domain of flagellin binds to a hydrophobic pocket within the LRR domain of NAIP5. This binding event triggers a conformational change in NAIP5, which in turn drives the oligomerization of NLRC4 and the activation of the functional inflammasome complex [65]. This interaction is highly specific: mutations in the D0 domain completely abolish NAIP5 binding and subsequent NLRC4 inflammasome activation, thereby markedly enhancing the intracellular survival capacity of *Salmonella* [66,67]. The specificity of this interaction may also represent an intrinsic host self-protection mechanism, which ensures that only bacterial flagellin, rather than endogenous host proteins, triggers NLRC4 inflammasome activation.

At the initial phase of host cell invasion by *Salmonella*, the T3SS-1 translocates flagellin into the host cell cytosol, which in turn drives a potent NLRC4 inflammasome response [26,68]. In addition to this canonical delivery pathway, a recent study has revealed that NLRC4-mediated recognition of *Salmonella* occurs independently of direct bacterial invasion. Mechanistically, *Salmonella* continuously releases outer membrane vesicles (OMVs) containing PAMPs, including flagellin and lipopolysaccharide (LPS). OMV-associated flagellin is translocated into the host cytosol through clathrin-mediated endocytosis, which triggers the NAIP5/NLRC4 axis independently of the T3SS apparatus [69]. Notably, OMV-mediated NLRC4 activation occurs much more rapidly than the non-canonical LPS/Caspase-11 pathway triggered by the same vesicles, indicating that NLRC4 acts as a “first responder” to bacterial products released during infection. Seminal studies have established that flagellin sensing by the NAIP5/NLRC4 inflammasome acts as a pivotal host defense axis against *Salmonella* infection. Genetic ablation of either NAIP5 or NLRC4 in mice results in drastically enhanced susceptibility to *Salmonella* challenge, as evidenced by elevated systemic bacterial loads and significantly impaired survival [33,70,71,72].

As a key virulence determinant of *Salmonella*, the T3SS is another critical target of the NLRC4 inflammasome, with its needle protein PrgI and inner rod protein PrgJ acting as the major ligands for NLRC4 activation [73]. The *Salmonella* T3SS, a needle-like complex spanning the bacterial inner and outer membranes as well as the host cell plasma membrane, directly translocates over 60 effector proteins into the host cell cytosol to facilitate bacterial manipulation of host cellular pathways and the establishment of a replicative intracellular niche [74]. T3SS-1 assembles during *Salmonella* host cell invasion, with PrgI forming the transmembrane channel and PrgJ constituting the channel base. Notably, the NLRC4 inflammasome cannot detect PrgI and PrgJ proteins when they are properly positioned within the intact T3SS apparatus [33]. Recognition only occurs when the T3SS translocates excess rod monomers from the bacterial cytosol or when rod monomers dissociate into the secretion channel, resulting in inadvertent translocation [20].

In mice, NAIP1 specifically recognizes PrgI, while NAIP2 is responsible for sensing PrgJ [75,76]. In humans, a single NAIP gene mediates recognition of both PrgI and PrgJ through alternative splicing of its mRNA (Figure 1) [22,26]. Accumulating evidence has established that NAIP-mediated recognition of PrgI and PrgJ is governed by conserved sequence motifs at the C-termini of both T3SS proteins, with the core binding sites for both located within a conserved hydrophobic helical hairpin structure at their C-terminal regions [23,38]. Ligand binding subsequently triggers a conformational change in NAIPs, initiating the assembly and activation of the NLRC4 inflammasome. Actually, the C-terminal domain of PrgJ shares structural homology with the C-terminal domain of flagellin, with complete sequence identity across the 7 amino acids at their extreme C-termini—a region essential for NLRC4 inflammasome activation. Point mutations within this domain, particularly in the key valine residue (V95 in PrgJ), completely abrogate NLRC4 inflammasome activation [20]. The structural similarity between PrgJ and flagellin provides the molecular basis for their recognition by related NAIPs [34,77]. This structural conservation likely arises from functional constraints imposed by their shared role in assembling hollow tubular structures while also creating a vulnerability that the host immune system exploits for pathogen detection. These conserved immune recognition epitopes inform the rational design of next-generation *Salmonella* vaccines. Retaining NLRC4-activating epitopes while abrogating virulence functions balances immunogenicity and safety, and optimized flagellin derivatives can also act as mucosal adjuvants to boost the efficacy of oral vaccines.

During *Salmonella* infection, both PrgJ and flagellin are actively translocated into the host cytosol, which may in turn trigger simultaneous activation of NAIP2 and NAIP5/6 (Figure 1). Biochemical and functional studies have demonstrated that concomitant cytosolic delivery of PrgJ and flagellin synergistically enhances NLRC4 inflammasome activation, resulting in markedly augmented Caspase-1 activation, pyroptosis, and proinflammatory cytokine secretion relative to stimulation with either ligand individually [23,37]. This synergistic effect is thought to arise from the ability of multiple NAIP-ligand complexes to nucleate NLRC4 oligomerization with greater efficiency. This cooperative sensing mechanism endows the host with a far more robust detection system for invasive bacterial pathogens. Should a pathogen downregulate the expression of any single ligand, the presence of other ligands remains sufficient to elicit full inflammasome activation. This built-in redundancy guarantees that the host can mount a potent and effective immune response against a diverse array of bacterial pathogens.

### 3.2. Positive Regulatory Network of the NLRC4 Inflammasome

Several protein regulators have been characterized to positively modulate NLRC4 inflammasome activation and host defense against *Salmonella* infection (Figure 1). Specifically, WD repeat-containing protein 90 (WDR90), a newly defined inflammasome component, directly engages NLRC4 to regulate its subcellular localization, thus licensing inflammasome assembly and activation [78]. Likewise, the vitamin D receptor (VDR) acts as an upstream positive regulator by directly binding to NLRC4 to facilitate stable assembly of the NAIPs-NLRC4 signaling complex [79]. Notably, autophagy related 16 like 2 (ATG16L2), an autophagy-related protein, exerts its pro-activating function via a distinct molecular axis: it directly associates with NAIPs, the upstream cytosolic sensors for bacterial ligands, to markedly potentiate ligand-induced NAIPs-NLRC4 complex formation and subsequent inflammasome activation [80]. Interferon regulatory factor 8 (IRF8) also targets NAIPs, but via a distinct mechanism: it directly binds to the promoter regions of *Naip* genes, thereby governing the transcription of NAIPs to enable detection of flagellin or T3SS components to mediate NLRC4 inflammasome activation [81,82]. These upstream positive regulators exert comprehensive regulatory control across transcription, protein–protein interactions, and subcellular localization, significantly enhancing both the sensitivity and magnitude of NLRC4 inflammasome activation and providing a critical early defense mechanism for the host to clear *Salmonella* infection. The functional redundancy generated by this multi-node regulatory mode, together with the multi-ligand cooperative recognition mechanism, collectively forms a highly robust immune defense system that effectively counteracts pathogen immune evasion strategies. The identification of these regulatory molecules not only refines our mechanistic understanding of NLRC4 inflammasome activation but also identifies potential therapeutic targets for developing novel host-directed anti-infective approaches, opening new avenues for tackling the increasingly severe global problem of antibiotic resistance.

### 3.3. Cell-Type-Specific Functions and Immune Crosstalk

The recognition of *Salmonella* by the NLRC4 inflammasome shows significant cell-type-specific differences, reflecting the diverse roles of different cell types in host defense. Intestinal epithelial cells (IECs) form the first line of defense against *Salmonella* infection. Upon *Salmonella* invasion of the intestinal tract, the NLRC4 inflammasome in IECs is rapidly activated. Instead of undergoing pyroptosis, infected IECs are extruded from the intestinal epithelium—a process that eliminates infected cells and prevents bacterial dissemination. Studies have shown that NAIP/NLRC4-deficient mice fail to expel infected IECs, leading to increased bacterial replication and systemic dissemination [27]. However, in human intestinal epithelial cells, NAIP/NLRC4 is not required for the early inflammasome response to *Salmonella*; human IECs mainly depend on Caspase-4 to mediate inflammasome activation in response to SPI-1-expressing *Salmonella* [83]. This interspecies difference in immune responses reveals an evolutionary divergence in intestinal innate immune defense mechanisms between mice and humans.

Macrophages are professional phagocytes that play a key role in host immunity against *Salmonella*. After *Salmonella* infection, the NLRC4 inflammasome in macrophages is rapidly activated, triggering pyroptosis and the release of pro-inflammatory cytokines [62]. This rapid pyroptosis eliminates the intracellular niche for bacterial replication and releases bacteria into the extracellular space, exposing them to the bactericidal effects of other immune cells [84,85]. Neutrophils are another important cell type involved in the early response to *Salmonella* infection. The NLRC4 inflammasome in neutrophils selectively promotes IL-1β maturation during acute *Salmonella* infection without inducing pyroptosis [86], allowing neutrophils to continue participating in the immune response and recruiting large numbers of immune cells to the infection site. In dendritic cells (DCs), NLRC4 recognizes *Salmonella* and induces IL-18 secretion, which directly stimulates memory CD8^+^ T cells to produce IFN-γ [87], indicating that DCs not only mediate antigen presentation but also regulate innate immune responses through inflammasome activation.

Beyond innate immune defense, NLRC4 inflammasome activation also plays an important regulatory role in adaptive immune responses. In intestinal DCs, NLRC4-mediated IL-18 secretion promotes the differentiation of Th1 and Th17 cells [88], which are essential for clearing systemic *Salmonella* infection. In addition, flagellin-induced NLRC4 activation enhances the antigen presentation ability of DCs and promotes the production of flagellin-specific CD4^+^ T cells and neutralizing antibodies [9,89,90], providing long-term protective immunity against reinfection. Collectively, the functional outputs of NLRC4 inflammasome activation differ drastically across cell types, forming a layered host defense network against *Salmonella* infection. Notably, this cell-type specificity also exhibits interspecies divergence. For instance, the reliance on NLRC4 for early intestinal defense differs between mice and humans [83]. This species-specific difference further complicates the translation of mouse-based findings to human mucosal immunity and represents an important unresolved issue in the field.

## 4. Molecular Strategies for *Salmonella* Evasion of NLRC4 Inflammasome Recognition and Activation

While the host has evolved a highly sophisticated NLRC4 inflammasome-mediated immune surveillance system to detect and eliminate *Salmonella*, this pathogen has counter-evolved an equally complex repertoire of immune evasion strategies to disrupt NLRC4 activation at multiple levels, thereby facilitating intracellular survival and persistent infection. These strategies are tightly regulated in a spatiotemporal manner to match the sequential stages of *Salmonella* infection, from intestinal invasion to systemic spread. Broadly, these evasion mechanisms fall into two distinct categories: indirect immune evasion, which enables bacterial immune stealth by reducing ligand availability or spatial separation from cytosolic sensors; and direct immune evasion, which actively targets core components of the NLRC4 signaling axis via secreted effector proteins to suppress inflammasome activation (Table 1).

### 4.1. Indirect Immune Evasion: Avoiding NLRC4 Recognition

Indirect evasion constitutes the primary immune stealth strategy of *Salmonella* during early intracellular colonization. It reduces the exposure of immunogenic ligands to cytosolic NLRC4 sensors via spatial separation, transcriptional downregulation or structural epitope alteration, without directly targeting inflammasome components.

#### 4.1.1. Spatial Sequestration Within SCVs

**Figure 2:**
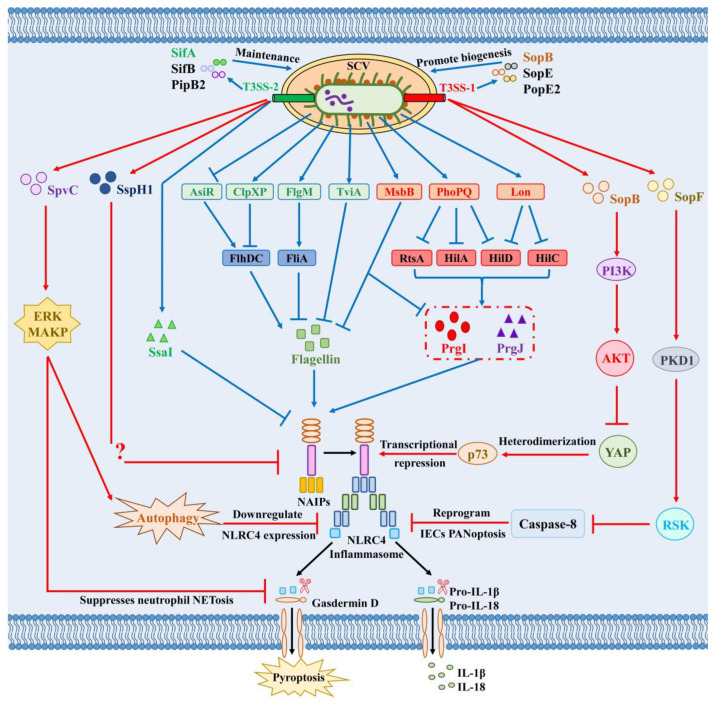
Multiple mechanisms by which *Salmonella* inhibits NLRC4 inflammasome activation. Indirect immune evasion pathways are highlighted in blue and direct immune evasion pathways in red. *Salmonella* forms SCVs via T3SS-1 effectors SopB, SopE, and SopE2, which serve as its primary intracellular replicative niche. Following host cell internalization, *Salmonella* switches its virulence program to rely on T3SS-2 effectors, including SifA, SifB, and PipB2, to maintain SCV homeostasis. Upon dissemination to systemic tissues, *Salmonella* rapidly downregulates T3SS-1 and flagellin expression while upregulating T3SS-2. This transcriptional rewiring represents a core immune evasion strategy that minimizes the production of NLRC4 inflammasome-activating ligands. *Salmonella* uses a multilayered regulatory network centered on the PhoP-PhoQ two-component system to silence T3SS-1 expression. PhoP directly represses the transcription of T3SS-1 regulators RtsA, HilA, and HilD, and simultaneously induces the Lon protease to degrade HilC and HilD, ensuring sustained T3SS-1 downregulation. For flagellar silencing, multiple complementary mechanisms are employed: the anti-sigma factor FlgM sequesters FliA to inhibit flagellar gene transcription; the acidic macrophage environment downregulates AsiR (an activator of *flhDC* expression) and induces ClpXP protease to degrade FlhDC; and the *Salmonella* Typhi-specific TviA directly represses flagellin expression. Together, these mechanisms enable *Salmonella* to rapidly shut down flagellar synthesis and evade NLRC4 recognition. In addition to ligand downregulation, *Salmonella* modifies its T3SS structure to avoid detection. The T3SS-2 inner rod protein SsaI contains multiple leucine-to-valine substitutions in its C-terminal NAIP recognition motif, which prevents its recognition by NAIP sensors. *Salmonella* also secretes T3SS effectors that directly inhibit NLRC4 inflammasome signaling. SopB activates the PI3K-Akt pathway, which disrupts the YAP-p73 heterodimer to repress *Nlrc4* transcription. SopF targets membrane phosphoinositides to activate the PDK1-RSK axis and inhibit Caspase-8, thereby suppressing GSDMD-mediated pyroptosis. The T3SS-2 effector SspH1 inhibits NLRC4 activation via E3 ubiquitin ligase activity (targets to be identified). SpvC inactivates the ERK MAPK pathway to impair autophagosome formation and downregulate NLRC4 expression, while also directly blocking NLRC4-Caspase-1-dependent GSDMD cleavage to suppress pyroptosis and IL-1β secretion. Figure 2 was independently designed by the authors using Microsoft PowerPoint 2021, with all elements created using native software features.

As an intracellular pathogen, *Salmonella* employs a highly effective and elaborate strategy to evade detection by most of the host immune system, including the NLRC4 inflammasome: it replicates and persists within SCVs, which are devoid of inflammasome activity. The T3SS-1 effector proteins SopB, SopE, and SopE2 orchestrate the biogenesis of the SCV during the early invasion stage of infection [91,92,93]. These effectors cooperatively induce massive actin cytoskeleton rearrangement in host cells, which drives membrane ruffling and triggers the formation of a membrane-bound SCV around internalized bacteria. Then T3SS-2 effector proteins, including SifA, SifB, and PipB2 mediate the maturation and maintenance of SCVs during the subsequent intracellular replication stage (Figure 2) [94,95,96,97,98]. They interact primarily with the host microtubule cytoskeleton to promote the formation of *Salmonella*-induced filaments and block SCV fusion with lysosomes and other degradative compartments. However, to achieve further systemic spread and colonize deeper systemic tissues, *Salmonella* must escape this permissive niche and exit the SCV [112,113]. At the same time, host cells use various mechanisms to target and disrupt SCVs, and their rupture re-exposes the bacteria to the bactericidal activity of the immune system [114].

#### 4.1.2. Transcriptional Silencing of Immunogenic Ligands

NLRC4 is the primary inflammasome activated during *Salmonella* infection, largely due to the abundant expression of flagellin and SPI-1-encoded T3SS-1 in logarithmic-phase *Salmonella*, which enables in vitro macrophages to rapidly recognize these bacteria within 1 h [115]. Although these conserved PAMPs are easily detected by the host, they play indispensable roles during bacterial invasion of epithelial cells [116]. However, when *Salmonella* enters the stationary phase and ceases expressing SPI-1, the NLRC4 inflammasome fails to be rapidly activated [20]. These findings indicate that the transcriptional program of *Salmonella* is essential for recognition by the NLRC4 inflammasome.

Unlike the NLRP3 inflammasome, the NLRC4 inflammasome responds to a highly restricted set of ligands. As a result, the evasion strategies adopted by *Salmonella* against NLRC4 inflammasome activation largely converge on flagellin and the T3SS-1. The PhoPQ two-component system plays a central role in regulating *Salmonella* gene expression in response to the intracellular environment. After breaching the intestinal epithelial barrier and disseminating to systemic tissues, *Salmonella* within the acidic environment of SCVs activates the PhoPQ system, which rapidly triggers downregulation of T3SS-1 expression and simultaneous upregulation of T3SS-2 expression [99]. This regulatory switch not only facilitates bacterial adaptation to the intracellular environment but also promotes immune evasion by reducing the expression of NLRC4-activating ligands, including PrgI/PrgJ and flagellin [117]. This PhoPQ-mediated SPI-1 silencing operates at three hierarchical regulatory levels: transcriptional repression, post-transcriptional silencing, and protein degradation. Mechanistically, PhoP exerts multilayered negative control over *hilA*, the gene encoding the main transcriptional regulator of T3SS structural genes: it directly binds to and represses the *hilA* promoter, indirectly inhibits transcription of the upstream master activators *hilD* and *rtsA*, and induces expression of the sRNA PinT for post-transcriptional silencing (Figure 2) [100]. Complementing this transcriptional regulation, *Salmonella* employs the Lon protease to target HilC and HilD for proteolytic degradation; this process is primarily triggered by intracellular stress signals such as acidic pH and nutrient limitation and constitutes a coordinated regulatory program enabling *Salmonella* to adapt to its intracellular parasitic niche, ensuring robust and sustained SPI-1 downregulation [101]. Small-molecule inhibitors targeting the PhoPQ regulatory axis can restore the expression of immunogenic bacterial ligands and re-enable NLRC4-mediated immune surveillance, representing a promising host-directed anti-infective strategy to combat drug-resistant *Salmonella*.

*Salmonella* has evolved numerous mechanisms to spatiotemporally regulate flagellin expression to balance the need for motility during invasion and the selective pressure of immune evasion. Among these, the anti-sigma factor FlgM plays a central role: it binds to FliA to inhibit transcription of class III flagellar genes, thus rapidly shutting down flagellar synthesis upon entry into systemic tissues and effectively evading recognition by the host NLRC4 inflammasome (Figure 2) [28]. Deletion of *flgM* leads to constitutive flagellin expression and markedly attenuates both systemic and mucosal *Salmonella* infections in an NLRC4 inflammasome-dependent manner. Furthermore, *Salmonella* harnesses the ClpXP ATP-dependent protease to specifically target the constitutively expressed flagellar master regulator FlhDC for proteolytic degradation, resulting in the rapid downregulation of flagellin expression [102,103,104]. The acidic pH environment in macrophages promotes downregulation of *Salmonella* AsiR, a putative RpiR family transcriptional regulator. AsiR directly binds to the *flhDC* promoter to promote flagellin expression (Figure 2). Downregulation of AsiR exerts a positive effect on *Salmonella* replication in macrophages and systemic infection in mice, as it effectively reduces flagellin-induced NLRC4 inflammasome activation and IL-1β release [105]. TviA, a transcription regulator uniquely encoded by *Salmonella* Typhi, has been demonstrated to specifically repress flagellin expression during human macrophage infection, thereby allowing the bacterium to evade NLRC4 inflammasome activation. Consistently, heterologous expression of the *tviA* gene in non-typhoidal *Salmonella* is sufficient to attenuate flagellin-induced NLRC4 inflammasome activation [63]. *Salmonella* also produce phase-variable flagellin, which enables them to switch between distinct flagellin variants. This mechanism allows *Salmonella* to evade the host immune response by expressing flagellin variants with reduced NAIP binding affinity [118].

Notably, a recent study showed that *Salmonella* does not immediately shut down flagellin expression upon macrophage invasion; instead, it gradually downregulates flagellin expression during infection. In the early phase of infection, lysophospholipids generated by NLRC4 inflammasome-dependent pyroptosis promote the release of monomeric flagellin from *Salmonella*. *Salmonella* infection induces TRIF-dependent type I interferon secretion, which in turn suppresses the expression of both NLRC4 and the lysophospholipid biosynthetic enzyme iPLA2, thereby inhibiting intracellular lysophospholipid production and ultimately downregulating flagellin expression. In NLRC4- or Caspase-1-deficient mice, as well as in mice with inhibited iPLA2 expression, the ability of *Salmonella* to downregulate flagellin is significantly enhanced, and bacterial burdens in the spleens of these mice are markedly increased [119]. Studies have revealed that *Salmonella* grown under SPI-2-inducing conditions that mimic the intracellular environment do not activate the NLRC4 inflammasome. However, forced expression of PrgJ or flagellin through an SPI-2 co-regulated promoter induces strong and sustained activation of the NLRC4 inflammasome in mice, resulting in rapid bacterial clearance from mouse organs [106]. These results indicate that inhibition of inflammasome activation is essential for *Salmonella* virulence and survival in the host. However, inflammasome activation is not exclusively harmful to bacteria. Studies have found that *fliC* transcription can be detected in bacteria isolated from Peyer’s patches of mice orally infected with *Salmonella*, but not in those isolated from mesenteric lymph nodes or spleens. In in vitro infection experiments, activation of *pagC* inhibits *fliC* transcription; however, in Peyer’s patches of infected mice, *fliC* is still transcribed under conditions where *pagC* is activated [102]. Forced expression of FliC in Peyer’s patches may play an important role in *Salmonella* pathogenesis. Flagellin activates both host inflammatory responses and FliC-specific immune responses, promoting bacterial spread to deep tissues. Subsequently, *Salmonella* represses *fliC* transcription in the new intracellular environment to evade recognition by the host NLRC4 inflammasome. A recent study showed that deletion of *Salmonella msbB*, which encodes an acyltransferase involved in the acylation of lipid A (a component of LPS), leads to lipid A underacylation. This not only reduces LPS endotoxicity but also downregulates the expression of FliC/FljB and PrgI/PrgJ, thereby decreasing NLRC4 inflammasome activation. These changes result in reduced bacterial virulence and increased sensitivity to antibiotics [120]. These results suggest that moderate induction of host inflammatory responses is necessary for successful *Salmonella* infection. In the long-term co-evolution with the host immune system, *Salmonella* maintains a delicate balance: it avoids rapid clearance by the host through moderate inhibition of NLRC4 inflammasome activation and disrupts the intracellular replication niche via limited inflammatory responses to promote further bacterial spread and systemic colonization in host tissues.

#### 4.1.3. Structural Modification of T3SS Apparatus

After internalization by host cells, *Salmonella* switches its primary secretion apparatus from T3SS-1 to T3SS-2, which is a critical prerequisite for its intracellular survival. The ability of flagellin and T3SS inner rod proteins to activate NAIP is closely related to their C-terminal regions, which are recognized by the LRR domains of NAIP [121]. While NAIP2 recognizes the T3SS-1 inner rod protein PrgJ, it cannot recognize the T3SS-2 inner rod protein SsaI (Figure 2). Compared with PrgJ, multiple leucine residues are replaced with valine residues in the C-terminal amino acid sequence of SsaI [106]. Structural and functional studies have shown that replacing the 8 C-terminal amino acid residues of PrgJ with the corresponding sequence from SsaI completely abolishes PrgJ’s ability to activate NLRC4. In contrast, replacing the C-terminal residues of SsaI with the corresponding residues from PrgJ allows the NLRC4 inflammasome to rapidly recognize SsaI, induces IL-1β secretion, and leads to rapid clearance of *Salmonella* [20]. The ability to switch between immunogenic and non-immunogenic T3SS systems represents a sophisticated immune evasion strategy that enables *Salmonella* to persist within host cells while minimizing detection by the NLRC4 inflammasome.

This immune evasion strategy, achieved by altering the recognition epitopes of key structural proteins, was long considered a pivotal mechanism by which T3SS-2 enables successful intracellular replication of *Salmonella*. However, a recent study demonstrated that while human NAIP similarly fails to recognize the T3SS-2 inner rod protein SsaI, it specifically recognizes the T3SS-2 needle protein SsaG (Figure 1) [89]. This finding overturns the long-standing paradigm that *Salmonella* T3SS-2 can completely evade detection by the NAIP/NLRC4 inflammasome. Notably, this species-specific recognition also explains why earlier mouse studies uniformly supported the T3SS-2 immune evasion dogma, while human cell studies have yielded conflicting results; the discrepancy stems from fundamental differences in the NAIP sensor system between the two species, rather than an experimental artifact. Even when human macrophages are infected with *Salmonella* mutant strains completely deficient in T3SS-1 and both flagellins (FliC and FljB), NAIP/NLRC4 can still induce IL-1β secretion and significantly restrict bacterial intracellular replication via recognition of SsaG. These data establish that this recognition mechanism retains its critical defensive function in the late stages of infection, when *Salmonella* has successfully downregulated T3SS-1 and flagellin expression. Mechanistically, SsaG retains a conserved C-terminal hydrophobic helical domain that is recognized by human NAIP, whereas murine NAIP lacks the ability to recognize this domain. This represents an important species-specific difference in *Salmonella* immune recognition between humans and mice, and may also be one of the key factors contributing to the disparities in *Salmonella* susceptibility and disease manifestations between the two species. This discrepancy also carries critical preclinical translation implications: conventional mouse models cannot fully recapitulate human NLRC4-mediated recognition of late-stage *Salmonella* infection, and humanized immune models or primary human cell validation are warranted to improve the translatability of NLRC4-targeted interventions.

### 4.2. Direct Immune Evasion: Active Inhibition of NLRC4 Signaling

Direct evasion dominates the mid-to-late stages of intracellular infection. *Salmonella* secretes T3SS effector proteins that directly target host signaling molecules or core inflammasome components, actively suppressing pathway activation even when immunogenic ligands are present.

#### 4.2.1. Transcriptional Repression of NLRC4 via Host Signaling Manipulation

Beyond the well-documented strategies to downregulate its own inflammasome ligands, *Salmonella* has evolved an additional layer of immune evasion by secreting effector proteins that interfere with NLRC4 inflammasome activation through both direct and indirect mechanisms. In B cells, *Salmonella* establishes infection via SPI-1-mediated pinocytosis, and its T3SS-1 effector SopB initiates a signaling cascade centered on phosphoinositide 3-kinase (PI3K). SopB-dependent PI3K activation drives the generation of phosphatidylinositol 3,4,5-trisphosphate (PIP3), which in turn recruits and activates protein kinase B (Akt). Activated Akt disrupts the cytoplasmic heterodimerization of Yes-associated protein (YAP) and transcription factor p73, leading to transcriptional repression of *Nlrc4* (Figure 2). This signaling axis ultimately prevents NLRC4 inflammasome assembly, Caspase-1 activation, and subsequent IL-1β secretion [107]. A separate study has corroborated these observations, demonstrating that SopB also activates Akt signaling in macrophages to suppress NLRC4 inflammasome activation. This inhibitory effect occurs through an ASC- and Caspase-1-dependent pathway and is strictly dependent on the intrinsic phosphatase activity of SopB [108].

#### 4.2.2. Effector-Mediated Interference with Inflammasome Signaling and Pyroptosis

Another T3SS-1 effector, SopF, has recently been identified to target phosphoinositide on the host cell membrane, activate the PDK1 (3-phosphoinositide-dependent protein kinase 1)-RSK (Ribosomal S6 kinase) signaling axis and inhibit Caspase-8 activation, thereby reprogramming IEC PANoptosis (an integrated form of programmed cell death that concurrently activates pyroptosis, apoptosis, and necroptosis) by suppressing apoptosis and GSDMD-mediated pyroptosis while promoting necroptosis [109]. Although its potential crosstalk with the NLRC4-Caspase-1 inflammasome pathway requires further validation, SopF clearly enables *Salmonella* to block infected epithelial cell extrusion, disrupt intestinal barrier function, attenuate mucosal inflammation, and promote systemic dissemination. This represents a critical innate immune evasion strategy employed by *Salmonella* during early mucosal infection.

For a long time, the functional characterization of the T3SS-2 has been largely restricted to the intracellular replication stage of *Salmonella* after macrophage invasion. However, recent studies have demonstrated that T3SS-2-mediated inhibition of host immune responses is detectable as early as 1 h post *Salmonella* infection, significantly earlier than the time of SCV rupture [111]. This indicates that T3SS-2 possesses an immune evasion function independent of its canonical role in maintaining SCV integrity. Notably, the inhibitory effect of T3SS-2 on the NLRC4 inflammasome is only observed in human macrophages but not in murine cells [111], suggesting that *Salmonella* has evolved host species-specific mechanisms to evade NLRC4 inflammasome recognition.

Notably, the selective pressure exerted by the NLRC4 inflammasome on *Salmonella* is not limited to the intracellular environment, but also extends to the intestinal luminal surface—a region traditionally regarded as a “blind spot” of innate immunity. A recent study has revealed a novel “chemotaxis-virulence synergy” immune evasion mechanism: *Salmonella* utilizes the chemotaxis receptor Tsr to migrate toward the nutrient-rich microenvironment adjacent to the intestinal epithelium [8], which is precisely the high immune pressure zone enriched with neutrophils following NLRC4 inflammasome activation. Only *Salmonella* strains harboring a functional T3SS-2 can survive in this zone, and the E3 ubiquitin ligase SspH1—encoded by the Gifsy3 prophage (a lysogenic prophage prevalent in wild-type *Salmonella* isolates) present in most wild-type isolates but absent from laboratory strain SL1344—plays a central role (Figure 2). Functional T3SS-2 inhibits NLRC4 inflammasome activation and the secretion of IL-1β and IL-18, thereby preventing excessive neutrophil recruitment and attenuating the phagocytic and bactericidal capacities of neutrophils [8], enabling *Salmonella* to successfully colonize this “dangerous yet fertile” microenvironment. This mechanism resolves a long-standing academic paradox: why *Salmonella* frequently accumulates loss-of-function mutations in the *tsr* gene in experimental evolution studies using SL1344 [122,123], whereas Tsr-mediated chemotaxis function is universally conserved in wild-type strains. When *Salmonella* lacks a fully functional T3SS-2 and thus cannot effectively modulate the NLRC4 inflammasome-mediated mucosal immune response, Tsr-driven chemotaxis that directs bacteria to the high immune pressure zone only accelerates bacterial death and clearance, leading to strong positive selection for *tsr* mutations.

SpvC, an effector protein encoded by the *Salmonella* virulence plasmid, is also one of the core molecules mediating T3SS-2-dependent immune evasion. While secreted in vitro by both T3SS-1 and T3SS-2, SpvC relies almost entirely on T3SS-2 for functional translocation and its major roles in intracellular infection and systemic virulence. Previous work has shown that SpvC uses its phosphothreonine lyase activity to specifically inhibit the ERK (Extracellular regulated protein kinases) MAPK (Mitogen-activated protein kinase) signaling pathway [31]. This inhibition impairs autophagosome formation in host cells, which in turn downregulates the protein expression levels of both NLRC4 and NLRP3, ultimately attenuating inflammasome activation and pyroptosis to promote bacterial intracellular survival. Recent studies from the same team have further revealed that SpvC acts in a cell-type-specific manner to inhibit GSDMD activation (Figure 2). In macrophages, SpvC blocks NLRC4-Caspase-1- and Caspase-11-dependent GSDMD cleavage to prevent pyroptosis and IL-1β secretion. In neutrophils, SpvC additionally suppresses GSDMD-mediated neutrophil NETosis (a neutrophil-specific form of cell death characterized by the release of DNA-containing extracellular traps to entrap and kill pathogens) [110]. This dual inhibitory activity disrupts the chemotactic crosstalk and functional synergy between macrophages and neutrophils, leading to attenuated intestinal inflammation and enhanced systemic bacterial dissemination.

Collectively, indirect and direct evasion strategies act in a spatiotemporally coordinated manner throughout the *Salmonella* infection cycle. Indirect stealth mechanisms predominate in the early intracellular colonization phase to minimize immune detection, while direct effector-mediated suppression takes effect in later stages to counteract activated host immune responses. This layered, sequentially regulated evasion network reflects the precise evolutionary adaptation of *Salmonella* to host innate immune pressure within the long-term co-evolutionary arms race.

## 5. Conclusions

The co-evolutionary arms race between *Salmonella* and the host innate immune system stands as a central determinant of infection outcomes, with the NLRC4 inflammasome forming the core battleground of this dynamic interplay. As a key cytosolic innate immune sensor, NLRC4 recognizes conserved *Salmonella* virulence determinants—flagellin and the T3SS apparatus—through the NAIP sensor family, representing a refined evolutionary adaptation of host innate immunity. The host tightly modulates NLRC4 activation at multiple levels to balance efficient pathogen clearance against excessive inflammatory tissue injury. The cell-type-specific functional outcomes of NLRC4 activation collectively form a layered defense network spanning mucosal barrier integrity and systemic immunity. Notably, the discovery that human NAIP recognizes the *Salmonella* T3SS-2 inner needle protein SsaG overturns the long-held dogma that T3SS-2 entirely evades NLRC4 detection, highlights critical species-specific differences between humans and mice, and carries important implications for translating findings from animal models to clinical settings. By integrating both sides of the host–pathogen interaction and systematically dissecting cross-species and cell-type-specific differences, this review provides a unified co-evolutionary framework that complements and extends existing descriptive summaries in the field.

Over the course of long-term co-evolution, *Salmonella* has evolved a multi-layered, spatiotemporally programmed immune evasion arsenal specifically targeting the NLRC4 axis, encompassing spatial sequestration in inflammasome-inactive compartments, transcriptional silencing of immunogenic ligands, structural remodeling of virulence machinery, and effector-mediated signaling interference. Each evasion strategy represents a precise adaptive response to host immune pressure, striking a balance between bacterial invasiveness and immune stealth. Despite substantial advances in the field, key mechanistic gaps persist, including the precise molecular targets of effectors such as SspH1 and the functional crosstalk between distinct evasion pathways.

Future investigations should prioritize two key directions: the heterogeneity of inflammasome signaling across tissues and distinct infection microenvironments and the structural basis of NAIP/NLRC4 ligand recognition—particularly the molecular mechanisms underpinning the broad ligand-sensing capacity of human NAIP. Cutting-edge approaches such as single-cell omics, spatial transcriptomics, and high-resolution live-cell imaging will be indispensable for dissecting the spatiotemporal dynamics of inflammasome assembly. A comprehensive mechanistic dissection of this host–pathogen arms race will deepen fundamental insights into innate immune regulation and pathogen evolution and provide a robust theoretical foundation for developing novel anti-infective therapeutics, next-generation *Salmonella* vaccines, and strategies to address the global challenge of antimicrobial resistance.

## Figures and Tables

**Table 1:** Summary of *Salmonella* immune evasion strategies targeting the NLRC4 inflammasome.

Category	Key Bacterial Factor	Specific Strategy	Host Target	Reference
Indirect evasion	SifA, SifB, SopB	Spatial sequestration in SCVs	Physical isolation (no direct target)	[91,92,93,94,95,96,97,98]
	PhoPQ system, Lon protease	Transcriptional silencing of T3SS-1 ligands	PrgI/PrgJ	[99,100,101]
	FlgM, ClpXP, AsiR, TviA	Transcriptional silencing of flagellin	FliC/FljB	[28,63,102,103,104,105]
	T3SS-2 encoded SsaI	T3SS structural epitope modification	NAIP ligand recognition domain	[20,106]
Direct evasion	SopB	Transcriptional repression of NLRC4	*Nlrc4* transcription (via PI3K-Akt-YAP axis)	[107,108]
	SopF	Inhibition of pyroptosis execution	Caspase-8/GSDMD	[109]
	SpvC	Inhibition of inflammasome signaling and GSDMD activation	ERK MAPK pathway/Autophagy/GSDMD	[31,110]
	SspH1 (T3SS-2 effector)	Mucosal immune suppression (target undefined)	NLRC4 signaling components (putative)	[8,111]

Abbreviations: SCV, *Salmonella*-containing vacuole; T3SS, type III secretion system; NLRC4, NLR family CARD-containing protein 4; PI3K, phosphoinositide 3-kinase; Akt, protein kinase B; YAP, Yes-associated protein; GSDMD, gasdermin D; ERK, extracellular regulated protein kinases; MAPK, mitogen-activated protein kinase.

## Data Availability

No new data were created or analyzed in this study. Data sharing is not applicable to this article.

## Abbreviations

The following abbreviations are used in this manuscript:

iNTS	Invasive non-typhoidal *Salmonella*
WHO	World Health Organization
NLRC4	NLR family CARD-containing protein 4
NAIP	NLR family apoptosis inhibitory protein
T3SS	Type III secretion system
SPI-1	*Salmonella* pathogenicity island-1
SPI-2	*Salmonella* pathogenicity island-2
SCV	*Salmonella*-containing vacuole
PRR	Pattern recognition receptor
PAMP	Pathogen-associated molecular pattern
NLR	NOD-like receptor
DAMP	Danger-associated molecular pattern
IPAF	ICE-protease activating factor
CARD	Caspase activation and recruitment domain
NACHT	Nucleotide-binding and oligomerization domain
LRR	Leucine-rich repeat
BIR	Baculoviral inhibitor of apoptosis protein repeat
ASC	Apoptosis-associated speck-like protein containing a CARD
PYD	Pyrin domain
GSDMD	Gasdermin D
GSDMD-NT	GSDMD N-terminal pore-forming domain
IL-1β	Interleukin-1β
IL-18	Interleukin-18
PKCδ	Protein kinase Cδ
LRRK2	Leucine-rich repeat kinase 2
SIRT3	Sirtuin 3
HUWE1	E3 ubiquitin ligase HUWE1
TRIM29	Tripartite motif containing 29
HERC2	E3 ubiquitin ligase HERC2
WDR90	WD repeat-containing protein 90
VDR	Vitamin D receptor
ATG16L2	Autophagy related 16 like 2
IRF8	Interferon regulatory factor 8
IECs	Intestinal epithelial cells
DCs	Dendritic cells
PI3K	Phosphoinositide 3-kinase
PIP3	Phosphatidylinositol 3,4,5-trisphosphate
Akt	Protein kinase B
YAP	Yes-associated protein
PDK1	3-phosphoinositide-dependent protein kinase 1
RSK	Ribosomal S6 kinase
Tsr	Chemotaxis receptor Tsr
ERK	Extracellular regulated protein kinases
MAPK	Mitogen-activated protein kinase
NETosis	Neutrophil extracellular trap formation
LPS	Lipopolysaccharide
OMVs	Outer membrane vesicles
